# Serine/threonine kinase-protein kinase B and extracellular signal-regulated kinase regulate ventilator-induced pulmonary fibrosis after bleomycin-induced acute lung injury: a prospective, controlled animal experiment

**DOI:** 10.1186/cc6983

**Published:** 2008-08-09

**Authors:** Li-Fu Li, Shuen-Kuei Liao, Chung-Chi Huang, Ming-Jui Hung, Deborah A Quinn

**Affiliations:** 1 Division of Pulmonary and Critical Care Medicine, Chang Gung Memorial Hospital, 5 Fu-Hsing Street, Kweishan, Taoyuan 333, Taiwan; 2 Chang Gung University, 259 Wen-Hwa 1st Road, Kweishan, Taoyuan 333, Taiwan; 3 Department of Respiratory Therapy, Chang Gung Memorial Hospital, 5 Fu-Hsing Street, Kweishan, Taoyuan 333, Taiwan; 4 Graduate Institute of Clinical Medical Sciences, Chang Gung University, 259 Wen-Hwa 1st Road, Kweishan, Taoyuan 333, Taiwan; 5 Cardiology Section, Department of Medicine, Chang Gung Memorial Hospital at Keelung, 222 Maijin Road, Keelung 204, Taiwan; 6 Pulmonary and Critical Care Unit, Department of Medicine, Massachusetts General Hospital, 55 Fruit Street, Bulfinch 148, Boston, MA 02114, USA; 7 Harvard Medical School, 25 Shattuck Street, Boston, MA 02115, USA; 8 Novartis Institute of Biomedical Research, 250 Massachusetts Avenue, Cambridge 02140, MA, USA

## Abstract

**Introduction:**

Lung fibrosis, reduced lung compliance, and severe hypoxemia found in patients with acute lung injury often result in a need for the support of mechanical ventilation. High-tidal-volume mechanical ventilation can increase lung damage and fibrogeneic activity but the mechanisms regulating the interaction between high tidal volume and lung fibrosis are unclear. We hypothesized that high-tidal-volume ventilation increased pulmonary fibrosis in acute lung injury via the serine/threonine kinase-protein kinase B (Akt) and mitogen-activated protein kinase pathways.

**Methods:**

After 5 days of bleomycin administration to simulate acute lung injury, male C57BL/6 mice, weighing 20 to 25 g, were exposed to either high-tidal-volume mechanical ventilation (30 ml/kg) or low-tidal-volume mechanical ventilation (6 ml/kg) with room air for 1 to 5 hours.

**Results:**

High-tidal-volume ventilation induced type I and type III procollagen mRNA expression, microvascular permeability, hydroxyproline content, Masson's trichrome staining, S100A4/fibroblast specific protein-1 staining, activation of Akt and extracellular signal-regulated kinase (ERK) 1/2, and production of macrophage inflammatory protein-2 and 10 kDa IFNγ-inducible protein in a dose-dependent manner. High-tidal-volume ventilation-induced lung fibrosis was attenuated in Akt-deficient mice and in mice with pharmacologic inhibition of ERK1/2 activity by PD98059.

**Conclusion:**

We conclude that high-tidal-volume ventilation-induced microvascular permeability, lung fibrosis, and chemokine production were dependent, in part, on activation of the Akt and ERK1/2 pathways.

## Introduction

Severe lung injuries are characterized by an initial diffuse inflammatory reaction or epithelial injury that is followed by fibroblast proliferation and extracellular matrix accumulation [1,2]. Death or long-term ventilator dependence after an episode of acute lung injury (ALI) is often a result of abnormal wound healing, characterized by overwhelming fibrosis, severe hypoxemia, and loss of lung compliance [3-5]. The factors that determine alveolar recovery or progressive fibrosis are unclear. Identification of the mechanisms regulating fibrosis may allow development of therapeutic targets for patients with lung fibrosis as a complication of ALI.

In acute respiratory distress syndrome (ARDS) – which is an inhomogeneous disease, with only a small portion of the lung compliant and ventilated – the potential for overdistension of more compliant areas of lung is great. The use of a high tidal volume in normal animals mimics this overdistension of normal lung. Although the ARDS Network trial demonstrated that low-tidal-volume ventilation is safer than high-tidal-volume ventilation, these findings have been questioned [6]. In the combined rat model of ventilator-induced lung injury (VILI) and acid aspiration, a tidal volume (V_T_) of 3 ml/kg was more protective than 6 ml/kg V_T_, so even a very low 6 ml/kg V_T _can still overdistend more compliant regions of the lung and cause lung injury [5]. High-tidal-volume ventilation has been shown to increase lung injury (VILI). VILI is characterized by noncardiogenic pulmonary edema and release of cytokines/chemokines [6,7]. In ALI there is an initial accumulation of neutrophils and a later loss of adhesion of epithelial cells to the basement membrane, which induces the epithelia to express inflammatory mediators such as macrophage inflammatory protein-2 (MIP-2) and 10 kDa IFNγ-inducible protein (IP-10) [8-11].

MIP-2 is a functional homolog of human IL-8 in rodents and has been shown to play a role in the pathogenesis of VILI [12,13]. Chemokines are expressed both in the acute inflammatory response and in the wound remodeling later after lung injury. In a previous study, MIP-2 also induced neovascularization and regulated angiogenesis/fibrosis during bleomycin-induced pulmonary fibrosis [8]. Blockade of MIP-2 significantly inhibits the angiogenetic activity and pulmonary fibrosis in bleomycin-treated lung specimens. IP-10 has been shown to regulate angiogenic activity in pulmonary fibrosis by limiting fibroblast migration [9]. IP-10 binds to CXC chemokine receptor 3 and is chemotactic for T cells and natural killer cells [10]. In animal studies, expression levels of the MIP-2 and IP-10 are inversely correlated with the degree of lung damage, with the extent of neovascularization, and with fibrogenesis in bleomycin-treated samples [11].

A previous study has shown that mitogen-activated protein kinases (MAPKs) may be associated with the regulation of inflammation and pulmonary fibrosis in ALI [14,15]. S100A4, a member of the S100 family of cytoplasmic proteins, is identical to fibroblast-specific protein 1 (FSP1), a lineage marker that uniquely identifies fibroblasts or epithelium undergoing epithelial–mesenchymal transition during tissue fibrogenesis [16,17]. The expression of S100A4/FSP1 in the epithelium indicates the presence of transition from epithelial cells to fibroblasts.

A previous study has shown that high-tidal-volume ventilation can lead to activation of serine/threonine kinase/protein kinase B (Akt) [18]. The significance of high-tidal-volume or low-tidal-volume ventilation in the formation of pulmonary fibrosis is unclear after ALI. To investigate the association between high-tidal-volume-induced neutrophil infiltration and different MAPK pathways and Akt, as well as the role of different MAPK pathways, extracellular signal-regulated kinase (ERK) 1/2 and p38, and Akt, we employed pharmacological inhibition and studies in Akt-deficient mice. We hypothesized that high-tidal-volume ventilation after bleomycin-induced ALI can increase lung fibrosis secondary to activation of the Akt and MAPK pathways.

## Materials and methods

### Experimental animals

Male C57BL/6 mice, either wild-type Akt^+/+ ^or Akt^+/- ^on a C57BL/6 background, aged between 6 and 8 weeks, weighing between 20 and 25 g, were obtained from Jackson Laboratories (Bar Harbor, ME, USA) and from the National Laboratory Animal Center (Taipei, Taiwan) as previously described [19]. The study was performed in accordance with animal experimental guidelines of the National Institutes of Health and with approval from the local research committee.

### Ventilator protocol

We used our established mouse model of VILI as previously described [13,20]. A 20-gauge angiocatheter was introduced into the tracheotomy orifice of the mouse under general anesthesia with intraperitoneal ketamine (90 mg/kg) and xylazine (10 mg/kg). The mice were placed in a supine position on a heating blanket and were then attached to a Harvard apparatus ventilator (model 55-7058; Harvard Apparatus, Holliston, MA, USA), set to deliver either 6 ml/kg at a rate of 135 breaths/minute or 30 ml/kg at a rate of 65 breaths/minute for 1 and 5 hours while breathing room air with zero end-expiratory pressure. The mice then received 0.9% saline containing maintenance ketamine (0.1 mg/g/hour) and xylazine (0.01 mg/g/hour) at a rate of 0.09 ml/10 g/hour by a continuous intraperitoneal fluid pump.

The tidal volume delivered by the ventilator was checked by fluid displacement from an inverted calibration cylinder. Continuous monitoring of end-tidal carbon dioxide by a microcapnograph (Columbus Instruments, Columbus, OH, USA) was performed. The respiratory frequencies of 135 breaths/minute for 6 ml/kg tidal volume and 65 breaths/minute for 30 ml/kg tidal volume were chosen in the experiment, with end-tidal carbon dioxide between 30 and 40 mmHg. The airway peak inspiratory pressure was measured with a pressure transducer amplifier (Gould Instrument Systems, Valley View, OH, USA) connected to the tubing at the proximal end of the tracheostomy. The mean arterial pressure was monitored every hour during mechanical ventilation using the same pressure transducer amplifier connected to a 0.61 mm outer diameter (0.28 mm inner diameter) polyethylene catheter ending in the common carotid artery.

At the end of the study period, heparinized blood was taken from the arterial line for analysis of arterial blood gas and the mice were sacrificed. Control, nonventilated mice were anesthetized and sacrificed immediately. The experimental design and the number of animals in the study are summarized in Table 1.

**Table 1:** Experimental design and numbers of animals per group

Mice	MIP-2, IP-10 (5 hours)	Hydroxyproline (5 hours)	Evans blue dye assay (5 hours)	Masson's trichrome stain (5 hours)	Immunohistochemistry^a ^(5 hours)	Western blot assay^b ^(1 hour)	RT-PCR (1 hour)
Control (without bleomycin)	6	6	6		6	6	6
Control (with bleomycin, 5 days)	6	6	6	6	6	6	6
6 ml/kg V_T _(with bleomycin, 5 days)	6	6	6	6	6	6	6
6 ml/kg V_T _(with bleomycin, 10 days)		6		6			
30 ml/kg V_T _(with DMSO)						6	
30 ml/kg V_T _(without DMSO)				6			
30 ml/kg V_T _(with bleomycin, 5 days)	6	6	6	6	6	6	6
30 ml/kg V_T _(with bleomycin, 10 days)		6		6			
30 ml/kg V_T _(with bleomycin, 5 days) + PD98059	6	6	6	6	6	6	6
V_T _30 ml/kg (with bleomycin, 10 days) + PD98059		6		6			
Control (with bleomycin, 5 days), Akt^+/-^					6		
30 ml/kg V_T _(with bleomycin, 5 days), Akt^+/-^	6	6	6	6	6	6	6
30 ml/kg V_T _(with bleomycin, 10 days), Akt^+/-^		6		6			
30 ml/kg V_T _(with bleomycin, 5 days) + SB203580	6	6	6	6	6	6	6

Time points for measurements were determined according to our previous findings that mediator activation occurs early in ventilator-induced lung injury, and neutrophil infiltration occurs later [20]. ^a^Serine/threonine kinase/protein kinase B, extracellular signal-regulated kinase 1/2, S100A4-positive. ^b^JNK, p38, extracellular signal-regulated kinase, serine/threonine kinase/protein kinase B. Control, spontaneously breathing, nonventilated mice; DMSO, dimethyl sulfoxide; IP-10, 10 kDa IFNγ-inducible protein; MIP-2, macrophage inflammatory protein-2 (a functional homologue of IL-8); V_T_, tidal volume; 5 hours and 1 hour, time of mechanical ventilation.

### Animals and bleomycin administration

The mice received a single dosage of 0.075 U bleomycin in 100 μl sterile normal saline (Sigma, St Louis, MO, USA) intratracheally, and were ventilated for 5 or 10 days after bleomycin administration [21].

### Pharmacological inhibitor

The P38 inhibitor (SB203580, 16 mg/kg; Calbiochem, La Jolla, CA, USA) and the ERK1/2 inhibitor (PD98059, 2 mg/kg; Calbiochem) were given subcutaneously 30 minutes before ventilation based on previous *in vivo *studies [22,23].

### Masson's trichrome stain and fibrosis scoring

The lung tissues from control, nonventilated mice exposed to high-tidal-volume ventilation or low-tidal-volume ventilation for 5 hours while breathing room air were paraffin embedded, sliced at 4 μm, deparaffinized, and stained sequentially with Weigert's iron hematoxylin solution, Biebrish scarlet-acid fuchsin solution, and aniline blue solution according to the manufacturer's instruction of a trichrome kit (Sigma). A blue signal indicated positive staining of collagen.

The fibrotic grade of each lung field was assessed using the criteria of Ashcroft, ranging from grade 0 to grade 5 as follows: grade 0 = normal lung; grade 1 = minimal fibrous thickening of alveolar or bronchial walls; grade 2 = moderate thickening of walls without obvious damage to the lung architecture; grade 3 = increased fibrosis with definite damage to the lung structure and formation of fibrous bands or small fibrous masses; grade 4 = severe distortion of the structure and large fibrous areas (honeycomb lung); and grade 5 = total fibrous obliteration in the field [14]. An average number of 10 nonoverlapping fields in Masson's trichrome staining of paraffin lung sections, six mice per group, were analyzed for each section by a single investigator blinded to the mouse genotype.

### Hydroxyproline assay

Lungs were homogenized in 2 ml PBS, and a 1 ml aliquot was desiccated and then hydrolyzed in 6 N HCl at 110°C for 12 hours. Twenty-five-microliter aliquots were added to 1 ml of 1.4% chloramine T (Sigma), 10% *n*-propanol, and 0.5 M sodium acetate, pH 6.0. After 20 min of incubation at room temperature, 1 ml Erlich's solution (1 M *p*-dimethylaminobenzaldehyde (Sigma) in 70% *n*-propanol, 20% perchloric acid) was added and a 15-minute incubation at 65°C was performed. Absorbance was measured at 550 nm and the amount of hydroxyproline was determined against a standard curve [21].

### Immunoblot analysis

The lungs were homogenized in 3 ml lysis buffer (20 mM HEPES, pH 7.4, 1% Triton X-100, 10% glycerol, 2 mM ethylene glycol-bis (β-aminoethyl ether)-*N*,*N*,*N'*,*N'*-tetraacetic acid, 50 μM β-glycerophosphate, 1 mM sodium orthovanadate, 1 mM dithiotreitol, 400 μM aprotinin, and 400 μM phenylmethylsulfonyl fluoride), were transferred to eppendorf tubes and were placed on ice for 15 minutes. The tubes were centrifuged at 14,000 rpm for 10 minutes at 4°C and the supernatant was flash frozen. Crude cell lysates were matched for protein concentration, resolved on a 10% bis-acrylamide gel, and electrotransferred to Immobilon-P membranes (Millipore Corp., Bedford, MA, USA).

For assay of phosphorylation of JNK, p38, ERK1/2, and Akt protein expression, western blot analysis were performed with antibodies to phospho-JNK, phospho-p38, phospho-ERK1/2, and phospho-Akt (New England BioLabs, Beverly, MA, USA). For determination of total JNK, p38, ERK1/2, and Akt protein expression, western blot analysis was performed with the respective antibodies (Santa Cruz Biotechnology, Santa Cruz, CA, USA). Blots were developed by enhanced chemiluminescence (NEN Life Science Products, Boston, MA, USA).

### Measurement of MIP-2 and IP-10

At the end of the study period, the lungs were lavaged via tracheostomy with a 20-gauge angiocatheter (sham instillation) three times with 0.6 ml of 0.9% normal saline. The effluents were pooled and centrifuged at 2,000 rpm for 10 minutes. Supernatants were frozen at -80°C for further analysis of cytokines. MIP-2 (1 pg/ml) and IP-10 (2.2 pg/ml) were measured in bronchoalveolar lavage fluid using a commercially available immunoassay kit containing antibodies cross-reactive with rat and mouse MIP-2 and IP-10 (Biosource International, Camarillo, CA, USA). Each sample was run in duplicate according to the manufacturer's instructions.

### Immunohistochemistry

The lung tissues from control, nonventilated mice exposed to high-tidal-volume ventilation or low-tidal-volume ventilation for 5 hours while breathing room air were removed *en bloc*, and were filled with 10% neutral buffered formalin (pH 6.8 to 7.2) at 30 cmH_2_O pressure via polyethylene tubing inserted into the trachea. The lungs were paraffin embedded, sliced at 4 μm, deparaffinized, antigen unmasked in 10 mM sodium citrate (pH 6.0), and incubated with phospho-Akt, phospho-ERK1/2 (1:100; New England BioLabs), S100A4 primary antibody (1:100; Thermo Fisher Scientific Anatomical Pathology, Fremont, CA, USA), and biotinylated goat anti-rabbit secondary antibody (1:100) of a immunohistochemical kit (Santa Cruz Biotechnology) according to the manufacturer's instructions. The specimens were further conjugated with horseradish peroxidase–streptoavidin complex, detected by diaminobenzidine substrate mixture, and counterstained by hematoxylin. A dark-brown diaminobenzidine signal indicated positive staining of phospho-Akt, phospho-ERK1/2, and S100A4 of epithelial cells or fibroblasts, while shades of light blue signified nonreactive cells.

### Evans blue dye analysis

Extravasation of Evans blue dye (Sigma Chemical) into the interstitium was used as a quantitative measure of changes of microvascular permeability in acute lung injury [13]. Thirty minutes before the end of mechanical ventilation, 30 mg/kg Evans blue dye was injected through the internal jugular vein. At the time of sacrifice after 5 hours of mechanical ventilation, the lungs were perfused free of blood with 1 ml of 0.9% normal saline via the right ventricle and removed *en bloc*. Evans blue dye was extracted from lung tissue after homogenization for 2 minutes in 5 ml formamide (Sigma Chemical) and was incubated at 37°C overnight. The supernatant was separated by centrifugation at 5,000 × *g *for 30 minutes, and the amount was recorded.

Evans blue dye in the plasma and lung tissue was quantitated by dual-wavelength spectrophotometric analysis at 620 nm and 740 nm. The method corrects the specimen's absorbance at 620 nm for the absorbance of contaminating heme pigments, using the following formula: corrected absorbance at 620 = actual absorbance at 620 nm - [1.426 (absorbance at 740) + 0.03]. We calculated the Evans blue dye amount extracted from lung tissue and divided the amount by the weight of lung tissue.

### Reverse transcription-polymerase chain reaction

Total RNA (1 μg) was reverse transcribed using a GeneAmp PCR system 9600 (PerkinElmer, Life Sciences, Inc., Boston, MA, USA), as previously described [20]. The following primers were used for PCR: type I procollagen, forward primer 5'-TGTGCCACTCTGACTGGAAGA-3' and reverse primer 5'-CAGACGGCTGAGTAGGGAACA-3'; type III procollagen, forward primer 5'-GGAAAGGATGGAGAGTCAGGAA-3' and reverse primer 5'-CATTGCGTCCATCAAAGCCT-3'; and GAPDH (internal control), forward primer 5'-AATGCATCCTGCACCACCAA-3' and reverse primer 5'-GTAGCCATATTCATTGTCATA-3' (Integrated DNA Technologies, Inc., Coralville, IA, USA) [24].

### Statistical evaluation

The western blots were quantitated using a National Institutes of Health image analyzer (Image J 1.27z; National Institute of Health, Bethesda, MD, USA) and are presented as the ratio of phospho-MAPK to MAPK or the ratio of phospho-Akt to Akt (relative phosphorylation) in arbitrary units. Values are expressed as the mean ± standard deviation of at least six experiments.

The data for Evans blue dye, hydroxyproline, MIP-2, and IP-10 were analyzed using Statview 5.0 (SAS Institute, Inc., Cary, NC, USA).

All results of the western blot analyses were normalized to control, nonventilated wild-type bleomycin-treated mice breathing room air. Analysis of variance was used to assess the statistical significance of the differences, followed by multiple comparisons with a Scheffe test. *P *< 0.05 was considered statistically significant.

## Results

### Physiologic data

There were no statistical differences in the pH, the arterial carbon dioxide pressure, and the mean arterial pressure at the beginning versus the end of mechanical ventilation (Table 2). High-tidal-volume ventilation was injurious, with more carbon dioxide production than that of the low-tidal-volume group, and this increased the arterial carbon dioxide pressure in the high-tidal-volume group.

**Table 2:** Physiologic conditions at the beginning and the end of ventilation

	Nonventilated room air	6 ml/kg tidal volume room air	30 ml/kg tidal volume room air
PH	7.40 ± 0.05	7.35 ± 0.02	7.33 ± 0.06
Arterial oxygen pressure (mmHg)	98.7 ± 0.6	82.3 ± 7.5	86.1 ± 1.3
Arterial carbon dioxide pressure (mmHg)	40.2 ± 0.5	39.1 ± 1.1	35.3 ± 1.9
Mean arterial pressure (mmHg)			
Start	86 ± 1.9	85.3 ± 3.0	84.6 ± 2.3
End	85 ± 0.8	81.3 ± 2.2	73.5 ± 7.1
Peak inspiratory pressure (mmHg)			
Start		9.5 ± 1.5	23.6 ± 2.6
End		11.7 ± 1.8	27.9 ± 3.8

Arterial blood gases and mean arterial pressure of normal nonventilated mice and of mice ventilated at tidal volumes of 6 ml/kg or 30 ml/kg for 5 hours (n = 10 per group).

### Inhibition of Akt/MAPK activation with Akt-deficient mice and pharmacological inhibitors

The inhibition of Akt/MAPK activation with Akt-deficient mice and pharmacological inhibitors reduced the high-tidal-volume-induced microvascular permeability, lung fibrosis, and chemokine production.

**Figure 1:**
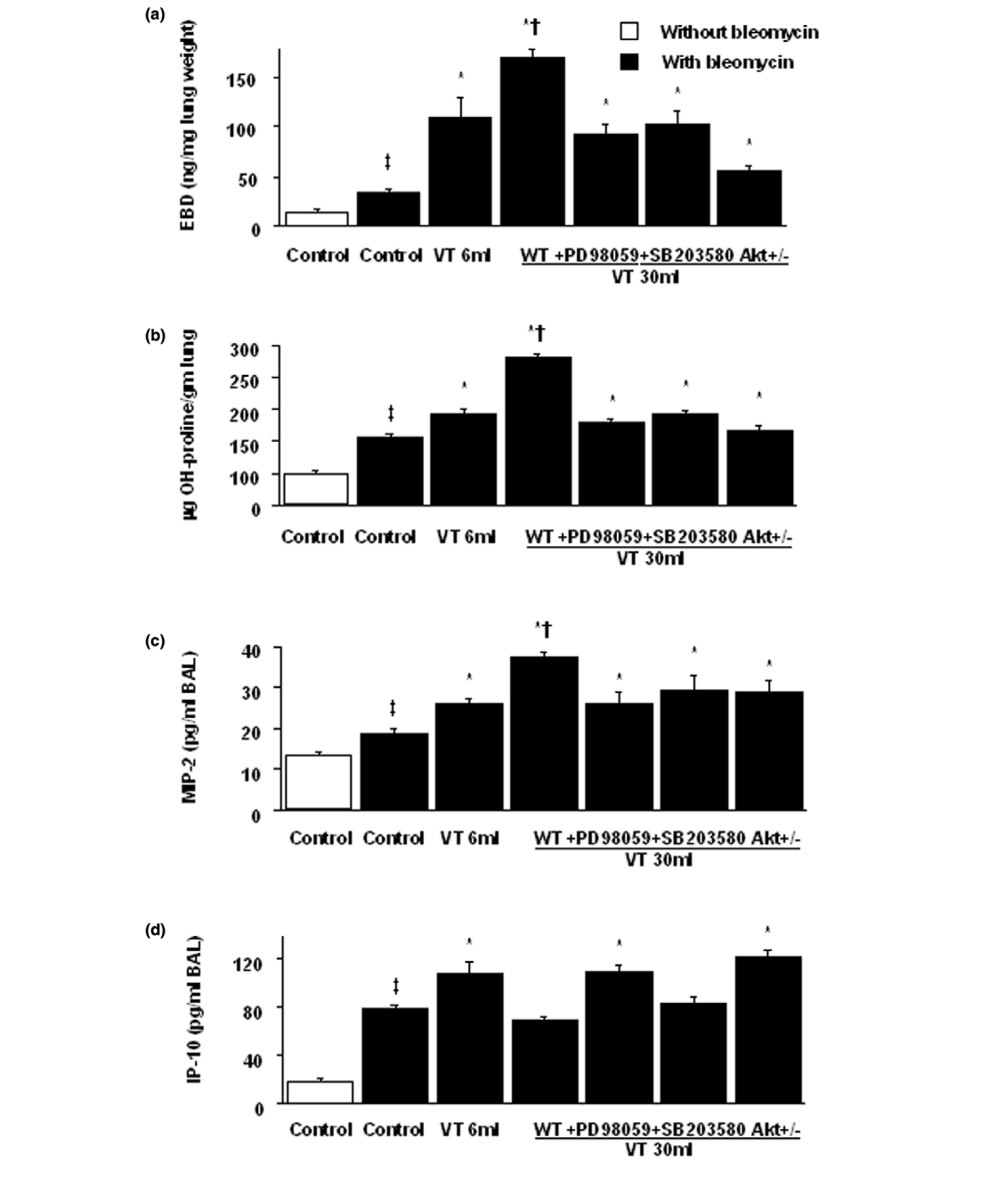
PD98059, SB203580, and Akt-deficient mice reduced stretch-induced microvascular leak, lung fibrosis, and chemokine production. After 5 days of bleomycin administration, wild-type C57BL/6 (WT) mice or serine/threonine kinase-protein kinase B (Akt)^+/- ^mice ventilated at a tidal volume (V_T_) of 6 ml/kg or 30 ml/kg for 5 hours were pretreated with 2 mg/kg PD98059 or 16 mg/kg SB203580 subcutaneously for 30 minutes. **(a) **Evans blue dye (EBD) assay and **(b) **the hydroxyproline (OH) content were obtained from lung tissues of mice (n = 6 per group). **(c) **Macrophage inflammatory protein-2 (MIP-2) production and **(d) **10 kDa IFNγ-inducible protein (IP-10) production were obtained from bronchoalveolar lavage (BAL) fluid of mice (n = 6 per group). *P < 0.05 versus control, nonventilated mice with bleomycin pretreatment; †*P *< 0.05 versus all other groups; ‡*P *< 0.05 versus control, nonventilated mice without bleomycin pretreatment.

In a previous study, we have shown that high-tidal-volume ventilation caused more pulmonary edema than in control, nonventilated rats or in rats ventilated at low tidal volume [25]. To measure the changes of microvascular permeability in VILI, we used the Evans blue dye assay (Figure 1a). The Evans blue dye levels significantly increased in mice receiving 30 ml/kg V_T _mechanical ventilation compared with those either of mice receiving 6 ml/kg V_T _or of control, nonventilated mice. The Evans blue dye levels were also significantly increased in 6 ml/kg V_T _mice compared with control, nonventilated mice.

In another previous study we showed that normalizing the EBD as nanograms per milligram of lung may have underestimated the amount of Evans blue dye for the high-tidal-volume group, but the data of Evans blue dye not normalized with lung weight showed similar results [26]. The data of hydroxyproline not normalized with lung weight showed a similar trend as those normalized with lung weight (control = 15.2 ± 0.9 μg; 6 ml/kg V_T _= 24.6 ± 2.8 μg, 30 ml/kg VT = 46.2 ± 1.6 μg, 30 ml/kg VT with PD98059 = 22.1 ± 3.1 μg, 30 ml/kg VT with SB203580 = 23.8 ± 1.7 μg, and 30 ml/kg VT in Akt^+/- ^mice = 18.4 ± 1.2 μg, all *P *< 0.05 versus control).

**Figure 2:**
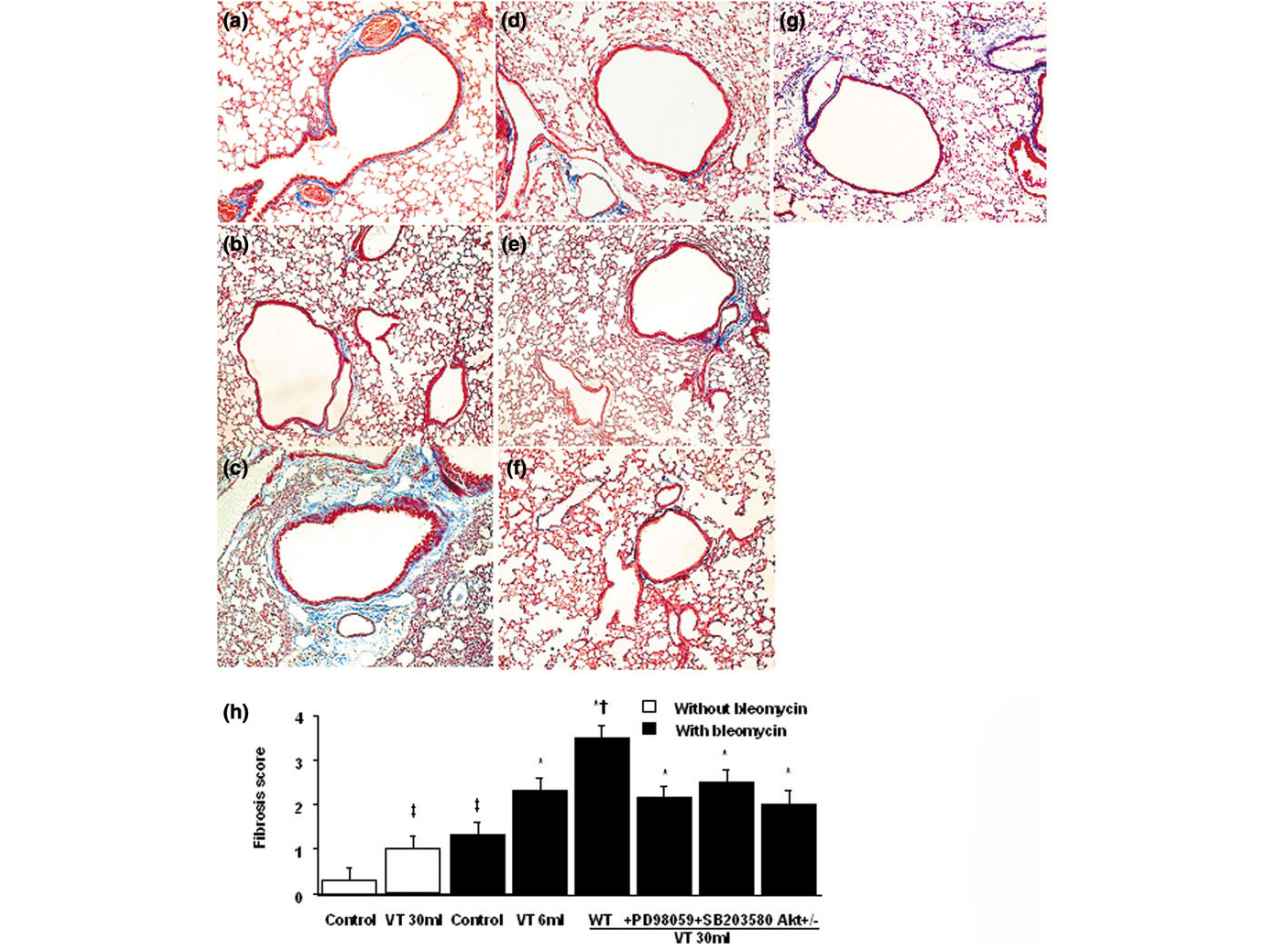
PD98059, SB203580, and Akt-deficient mice reduced high-tidal-volume-induced lung fibrosis. Representative photomicrographs (×100) with Masson's trichrome staining of paraffin lung sections 5 days after bleomycin-treatment in wild-type (WT) mice or serine/threonine kinase-protein kinase B (Akt)^+/- ^mice ventilated at a tidal volume (V_T_) of 6 ml/kg or 30 ml/kg for 5 hours with or without pretreatment with 2 mg/kg PD98059 or 16 mg/kg SB203580 subcutaneously for 30 minutes (n = 6 per group). **(a) **Control WT mice. **(b) **6 ml/kg V_T _WT mice. **(c) **30 ml/kg V_T _WT mice. **(d) **30 ml/kg V_T _WT mice pretreated with PD98059. **(e) **30 ml/kg V_T _WT mice pretreated with SB203580. **(f) **30 ml/kg V_T _Akt^+/- ^mice. Peribronchiolar and parenchymal blue staining indicates positive staining for lung fibrosis. **(g) **30 ml/kg V_T _WT mice without pretreatment with bleomycin. **(h) **Fibrotic scoring was quantified as the average number of 10 nonoverlapping fields in Masson's trichrome staining of paraffin lung sections (n = 6 per group). *P < 0.05 versus control, nonventilated mice with bleomycin pretreatment; †*P *< 0.05 versus all other groups; ‡*P *< 0.05 versus control, nonventilated mice without bleomycin pretreatment.

**Figure 3:**
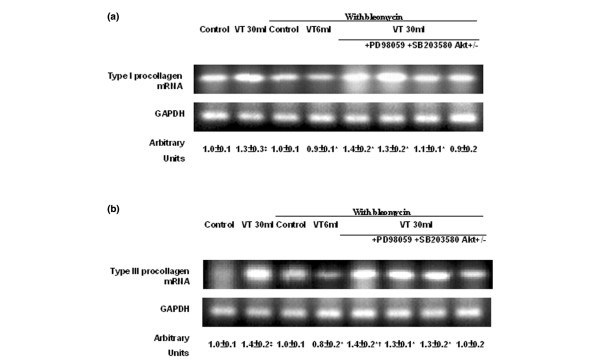
PD98059, SB203580, and Akt-deficient mice reduced high-tidal-volume-induced type III procollagen mRNA expression. Five days after bleomycin administration, wild-type mice or serine/threonine kinase-protein kinase B (Akt)^+/- ^mice ventilated at a tidal volume (V_T_) of 6 ml/kg or 30 ml/kg for 1 hour were pretreated with 2 mg/kg PD98059 or 16 mg/kg SB203580 subcutaneously for 30 minutes. RT-PCR assay was performed for **(a) **type I and **(b) **type III procollagen mRNA (top panel), GAPDH mRNA (middle panel), and arbitrary units (bottom panel) (n = 6 per group). Arbitrary units expressed as the ratios of type I and type III procollagen mRNA to GAPDH. *P < 0.05 versus control, nonventilated mice with bleomycin pretreatment; †*P *< 0.05 versus all other groups; ‡*P *< 0.05 versus control, nonventilated mice without bleomycin pretreatment.

To determine the effects of high-tidal-volume ventilation on pulmonary fibrosis, we measured the lung hydroxyproline content (Figure 1b) and performed Masson's trichrome staining and fibrosis scoring (Figure 2a,b,c,g). Five days after bleomycin treatment, an increase of peribronchiolar fibrosis and of parenchymal fibrosis was found in control nonventilated mice. The extent of fibrosis in mice ventilated at 30 ml/kg V_T _was significantly elevated compared with control, nonventilated mice, and compared with mice ventilated at 6 ml/kg V_T_. To determine the effects of high-tidal-volume ventilation on pulmonary fibrosis – which were measured by hydroxyproline content and Masson's trichrome staining, and were associated with upregulation of procollagen peptide – we measured type I and type III procollagen mRNA (Figure 3). Increases of type I and type III procollagen mRNA expressions were found in the 30 ml/kg V_T _mice compared with those of control, nonventilated mice, of 6 ml/kg V_T _mice, or of Akt-deficient mice.

**Figure 4:**
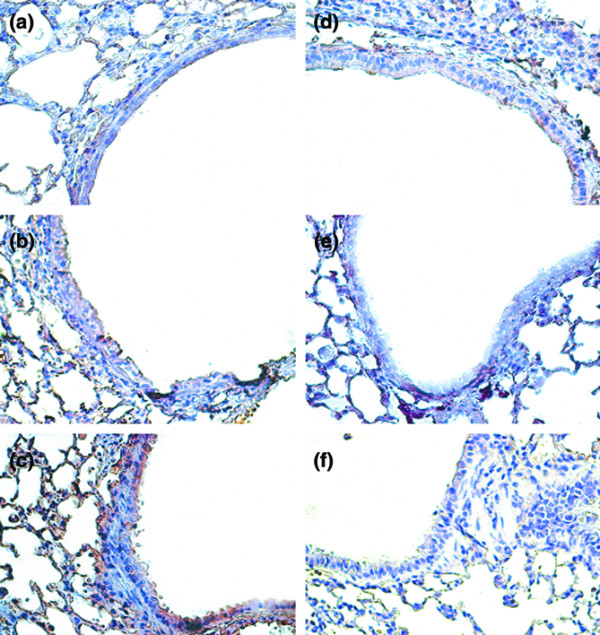
PD98059, SB203580, and Akt-deficient mice reduced high-tidal-volume-induced S100A4-positive fibroblast accumulation. Representative photomicrographs (×400) with S100A4 staining of paraffin lung sections via immunohistochemistry from 5 days of bleomycin treatment in wild-type (WT) mice or serine/threonine kinase-protein kinase B (Akt)^+/- ^mice ventilated at a tidal volume (V_T_) of 6 ml/kg or 30 ml/kg for 5 hours with or without pretreatment with 2 mg/kg PD98059 or 16 mg/kg SB203580 subcutaneously for 30 minutes (n = 6 per group). **(a) **Control WT mice. **(b) **6 ml/kg V_T _WT mice, **(c) **30 ml/kg V_T _WT mice. **(d) **30 ml/kg V_T _WT mice pretreated with PD98059. **(e) **30 ml/kg V_T _WT mice pretreated with SB203580. **(f) **30 ml/kg V_T _Akt^+/- ^mice. A dark-brown diaminobenzidine signal indicates positive staining for S100A4 in the lung epithelium or interstitium, while shades of bluish tan signify nonreactive cells.

To further define the cells types involved in the lung stretch-induced fibrogenesis, we measured S100A4/FSP1 using immunohistochemistry (Figure 4). The increased positive staining of S100A4/FSP1 in the epithelium of mice ventilated at 30 ml/kg V_T _compared with that of control, nonventilated mice and of mice ventilated at 6 ml/kg V_T _indicated the presence of a transition from epithelial cells to fibroblasts.

To explore the chemoattractants in VILI we measured MIP-2 and IP-10, involved in angiogenetic activity and angiostatic activity, respectively (Figure 1c,d). Increased production of MIP-2 but reduced production of IP-10 was found in mice ventilated at 30 ml/kg V_T _compared with control, nonventilated mice and compared with mice ventilated at 6 ml/kg V_T_. The mechanism regulating the increased chemokine production involved in pulmonary fibrosis needs to be further explored.

**Figure 5:**
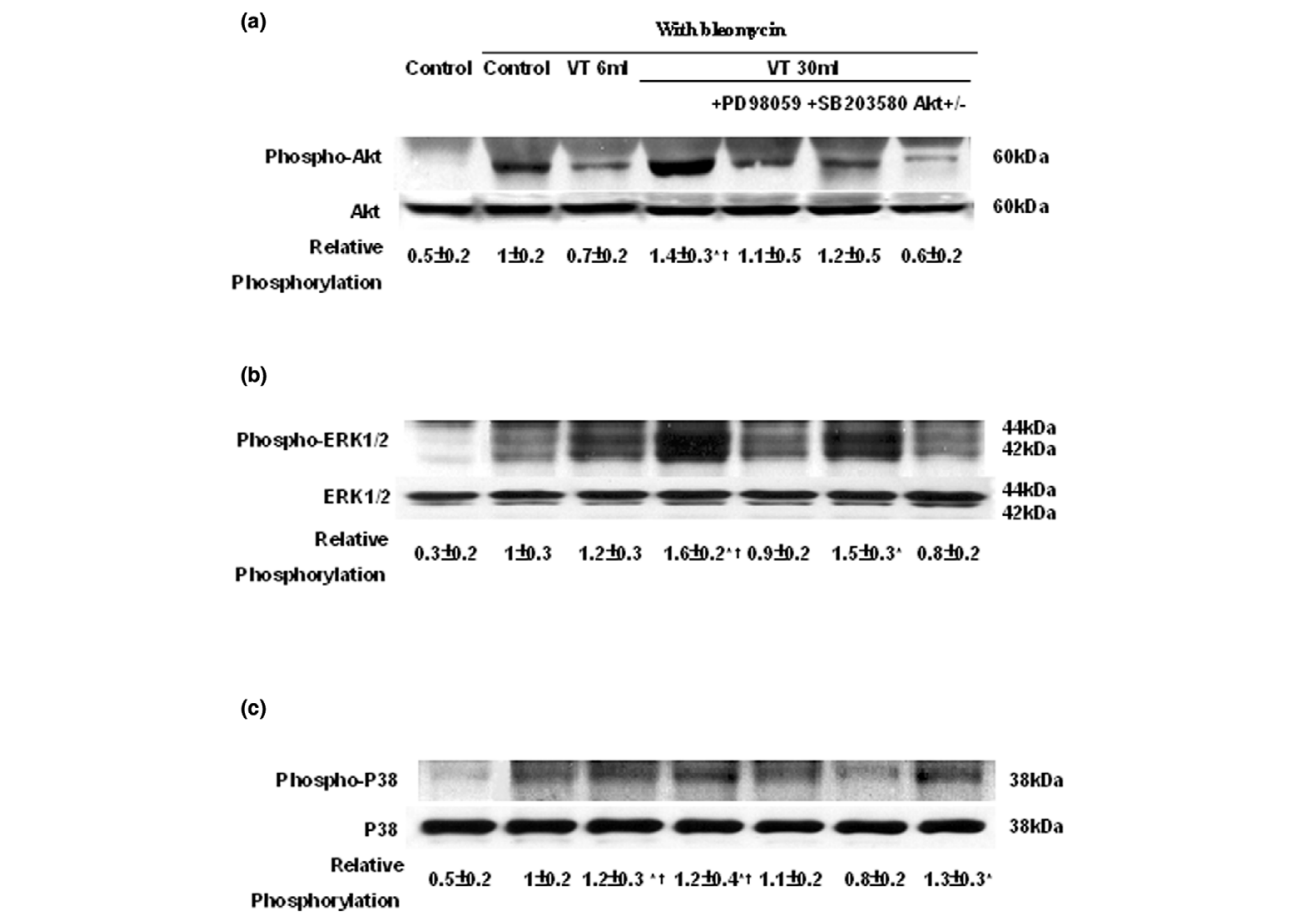
PD98059, SB203580, and Akt-deficient mice reduced stretch-induced Akt and mitogen-activated protein kinase activation. After 5 days of bleomycin administration, wild-type mice or serine/threonine kinase-protein kinase B (Akt)^+/- ^mice ventilated at a tidal volume of 6 ml/kg or 30 ml/kg for 1 hour were pretreated with 2 mg/kg PD98059 or 16 mg/kg SB203580 subcutaneously for 30 minutes. Western blot analysis was performed using an antibody that recognizes phosphorylated Akt expression, extracellular signal-regulated kinase (ERK) 1/2 expression, or P38 expression (**(a) **to **(c) **top panel), and an antibody that recognizes total Akt, ERK1/2, or P38 protein expressions in lung tissue ((a) to (c) middle panel). Arbitrary units expressed as relative Akt, ERK1/2, or P38 phosphorylation ((a) to (c) bottom panel) (n = 6 per group). *P < 0.05 versus control, nonventilated mice with bleomycin pretreatment; †*P *< 0.05 versus all other groups; ‡*P *< 0.05 versus control, nonventilated mice without bleomycin pretreatment.

We have previously shown that high-tidal-volume ventilation induced neutrophil infiltration via MAPK pathways [20]. We measured the activity of Akt and three members of the MAPK families – JNKs, p38, and ERK1/2 – to determine the stretch-induced kinase phosphorylation using 6 ml/kg V_T _and 30 ml/kg V_T _mechanical ventilation (Figure 5). There were dose-dependent increases in phosphorylation of Akt and ERK1/2 but there were no significant changes in the expression of total nonphosphorylated proteins of Akt and ERK1/2. Increased phosphorylation of P38 after either 6 ml/kg V_T _or 30 ml/kg V_T _mechanical ventilation occurred, but no significant changes in the expression of total nonphosphorylated protein P38 were found. There was no significant difference between 6 ml/kg V_T _mice or 30 ml/kg V_T _mice.

The phosphorylation of Akt, ERK1/2, and P38 was further increased after 5 hours of mechanical ventilation (relative phosphorylation of Akt: control = 1.0 ± 0.1; 30 ml/kg V_T_, 1 hour = 1.4 ± 0.3* and 30 ml/kg V_T_, 5 hours = 1.6 ± 0.4*; relative phosphorylation of ERK1/2: control = 1.0 ± 0.2; 30 ml/kg V_T_, 1 hour = 1.6 ± 0.2* and 30 ml/kg V_T_, 5 hours = 1.7 ± 0.3*; relative phosphorylation of p38: control = 1.0 ± 0.1; 30 ml/kg V_T_, 1 hour = 1.2 ± 0.3* and 30 ml/kg V_T_, 5 hours = 1.5 ± 0.4*; *P < 0.05 versus control). No significant increases of phosphorylation of JNK were found between control, nonventilated mice and mice ventilated at either 6 ml/kg V_T _or 30 ml/kg V_T _(control = 1.0 ± 0.2; 6 ml/kg V_T _= 1.1 ± 0.3 and 30 ml/kg V_T _= 0.9 ± 0.2, *P *= 0.12 versus control). The roles of Akt, ERK1/2, and P38 in the regulation of high-tidal-volume ventilation during lung fibrosis need to be further explored.

To determine the roles of Akt, EKR1/2, and P38 activation in stretch-induced lung fibrosis, we used Akt-deficient mice and pharmacological inhibitors of ERK1/2 and P38 (Figures 1 to 5). The type III procollagen mRNA expression, the microvascular permeability, the quantitative and qualitative evaluation of pulmonary fibrosis by hydroxyproline assay and Masson's trichrome staining, the positive S100A4/FSP1 staining of epithelium and fibroblasts in the interstitium, and the phosphorylation of Akt and ERK1/2 were significantly reduced after using Akt-deficient mice and pharmacological inhibition with PD98059 and SB203580. Reduced production of MIP-2 but increased production of IP-10 was found after using Akt-deficient mice and pharmacological inhibition with PD98059. Furthermore, PD98059 did not significantly decrease phosphorylation of Akt, but Akt-deficient mice reduced the phosphorylation of ERK1/2, suggesting that the Akt–ERK1/2 pathway was involved in the regulation of stretch-induced pulmonary fibrosis. Pharmacological inhibition with SB203580 significantly reduced the permeability, the lung fibrosis staining of collagen and fibroblasts, the phosphorylation of P38, and MIP-2 production, but not IP-10 production – suggesting that P38 played a less significant role than the Akt–ERK1/2 pathway in the mechanism of high-tidal-volume-induced pulmonary fibrosis.

**Figure 6:**
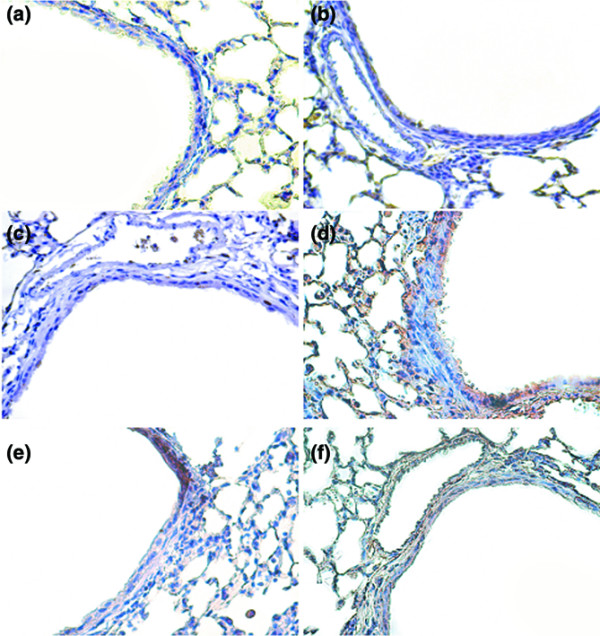
Akt-deficient mice reduced high-tidal-volume-induced Akt activation. Representative photomicrographs (×400) with phospho-serine/threonine kinase-protein kinase B staining of lung sections via immunohistochemistry after 5 days in bleomycin-treated wild-type (WT) mice or serine/threonine kinase-protein kinase B (Akt)^+/- ^mice ventilated at a tidal volume (V_T_) of 6 ml/kg or 30 ml/kg for 5 hours with or without pretreatment with 2 mg/kg PD98059 subcutaneously for 30 minutes (n = 6 per group). **(a) **Control WT mice. **(b) **Control Akt^+/- ^mice. **(c) **6 ml/kg V_T _WT mice. **(d) **30 ml/kg V_T _WT mice. **(e) **30 ml/kg V_T _WT mice pretreated with PD98059. **(f) **30 ml/kg V_T _Akt^+/- ^mice. A dark-brown diaminobenzidine signal indicates positive staining for S100A4 in the lung epithelium or interstitium, while shades of bluish tan signify nonreactive cells.

**Figure 7:**
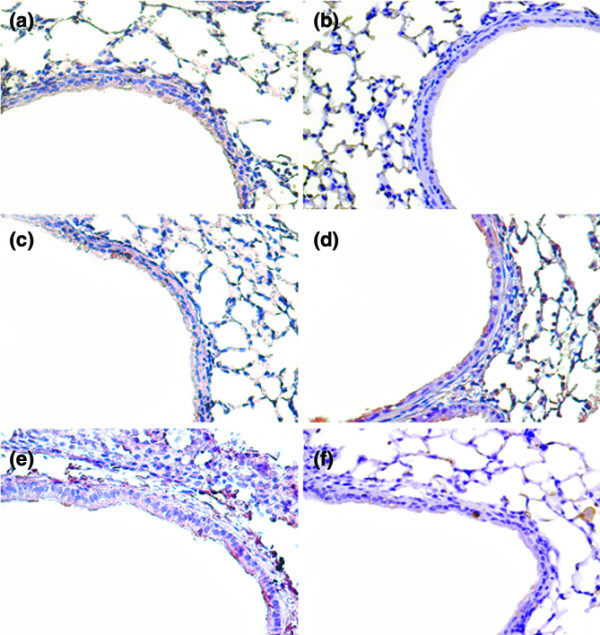
PD98059 and Akt-deficient mice reduced high-tidal-volume-induced ERK1/2 activation. Representative photomicrographs (×400) with phospho-extracellular signal-regulated kinase (phospho-ERK1/2) staining of lung sections via immunohistochemistry after 5 days of bleomycin treatment in wild-type (WT) mice or serine/threonine kinase-protein kinase B (Akt)^+/- ^mice ventilated at a tidal volume (V_T_) of 6 ml/kg or 30 ml/kg for 5 hours with or without pretreatment with 2 mg/kg PD98059 subcutaneously for 30 minutes (n = 6 per group). **(a) **Control WT mice. **(b) **Control Akt^+/- ^mice. **(c) **6 ml/kg V_T _WT mice. **(d) **30 ml/kg V_T _WT mice. **(e) **30 ml/kg V_T _WT mice pretreated with PD98059. **(f) **30 ml/kg V_T _Akt^+/- ^mice. A dark-brown diaminobenzidine signal indicates positive staining for S100A4 in the lung epithelium or interstitium, while shades of bluish tan signify nonreactive cells.

There were no statistically significant differences between phosphorylation of Akt and ERK1/2 in bleomycin-treated mice exposed to 1 hour of 30 ml/kg V_T _mechanical ventilation with or without pretreatment with vehicle (dimethyl sulfoxide) subcutaneously for 30 minutes (relative phosphorylation: control = 1.0 ± 0.1, Akt with vehicle = 1.37 ± 0.13 and Akt without vehicle = 1.42 ± 0.24, both *P *< 0.05 versus control; and control = 1.0 ± 0.11, ERK1/2 with vehicle = 1.51 ± 0.15 and ERK1/2 without vehicle = 1.64 ± 0.19, both *P *< 0.05 versus control). Using immunohistochemistry, we confirmed that high-tidal-volume ventilation induced Akt and ERK1/2 activation in bronchial epithelial cells (Figures 6 and 7).

**Figure 8:**
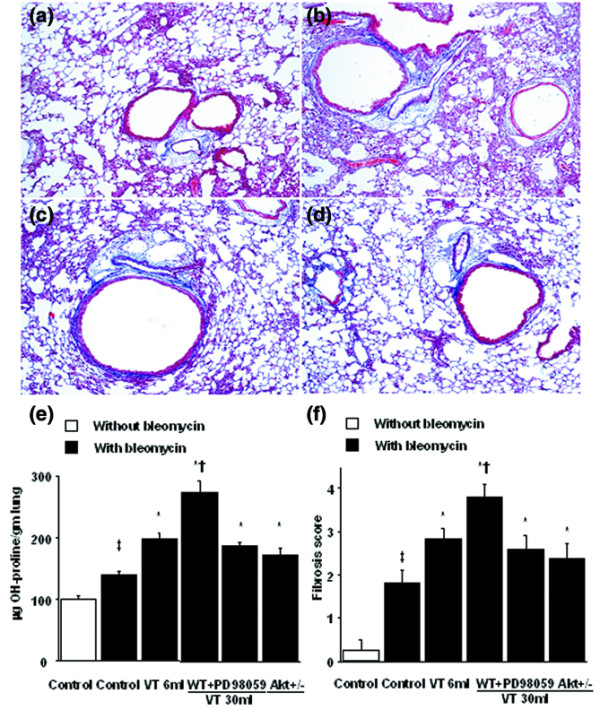
PD98059, and Akt-deficient mice reduced high-tidal-volume-induced lung fibrosis. Representative photomicrographs (×100) with Masson's trichrome staining of paraffin lung sections obtained after 10 days of bleomycin treatment in wild-type (WT) mice or serine/threonine kinase-protein kinase B (Akt)^+/- ^mice ventilated at a tidal volume (V_T_) of 6 ml/kg or 30 ml/kg for 5 hours with or without pretreatment with 2 mg/kg PD98059 subcutaneously for 30 minutes (n = 6 per group). **(a) **6 ml/kg V_T _WT mice. **(b) **30 ml/kg V_T _WT mice. **(c) **30 ml/kg V_T _WT mice pretreated with PD98059. **(d) **30 ml/kg V_T _Akt^+/- ^mice. Peribronchiolar and parenchymal blue staining indicates positive staining for lung fibrosis. **(e) **Hydroxyproline (OH) contents were obtained from lung tissues of mice (n = 6 per group). **(f) **Fibrotic scoring was quantified as the average number of 10 nonoverlapping fields in Masson's trichrome staining of paraffin lung sections (n = 6 per group). *P < 0.05 versus control, nonventilated mice with bleomycin pretreatment; †*P *< 0.05 versus all other groups; ‡*P *< 0.05 versus control, nonventilated mice without bleomycin pretreatment.

To determine whether the Akt–ERK1/2 pathway was involved in the late fibroproliferative phase of VILI, mice exposed to 10 days of bleomycin administration were ventilated. Similar trends involving the Akt–ERK1/2 signaling pathway were found with higher grades of lung fibrosis (Figure 8).

## Discussion

Bleomycin exposure results in an acute inflammatory reaction followed by pulmonary fibrosis that slowly resolves [27-29]. Bleomycin cleavage of DNA progresses from initial inflammation to final fibrosis [2]. Severe ALI can progress to the more severe form (ARDS). After about 1 week of lung injury, the fibroproliferative phase begins [30] – similar to what is seen in the bleomycin model. Death during the fibroproliferative phase of ARDS is the result of overwhelming pulmonary fibrosis-related reduced pulmonary compliance and severe hypoxemia. Identification of the mechanisms regulating the fibroproliferative phase of ARDS may help in the development of better treatment for pulmonary fibrosis in ARDS patients. In this mouse model of ALI from bleomycin exposure, we found that high-tidal-volume ventilation increased the microvascular permeability, the hydroxyproline content, Masson trichrome staining, positive staining of S100A4/FSP1 in the epithelium and interstitial fibroblasts, and production of MIP-2, but reduced production of IP-10. Akt–ERK1/2 pathways regulated the increase of lung fibrosis (Figures 1 to 8).

The predominant cell types involved in pulmonary fibrosis are fibroblasts and myofibroblasts, and the damaged epithelium can activate transformation of fibroblasts to myofibroblasts or can activate fibroblast proliferation through the secretion of cytokines [2]. Fibroblasts synthesize an extracellular matrix comprising collagen type I and type III, fibronectin, and proteoglycans [17]. Fibroblast proliferation and extracellular matrix synthesis are initiated 4 to 14 days post bleomycin challenge [8]. In a previous study, histopathologic evidence of fibroproliferation was found as early as 5 days in the course of ARDS [31]. Five days of bleomycin administration was thus used in our study to focus on the major target involved in the early phase of pulmonary fibrosis after ALI. In our study, we also found there were similar signaling pathways involved in the early (5 days) and late (10 days) phase of ALI with high-tidal-volume ventilation (Figure 8). No progressive rise in collagen content in the late phase of ALI may be due to functional recovery of underlying pulmonary disorder [11].

A high tidal volume increased lung fibrosis, as found by qualitative detection of peribronchiolar and parenchymal fibrosis via Masson's trichrome stain and fibrosis scoring, quantitative measurement of the collagen level via hydroxyproline assay, and FSP1 staining for fibroblasts via immunohistochemistry (Figures 1b, 2, and 4). In the course of ARDS, lung inflammation and fibrosis to some degree interact. It is important to be careful in differentiating the effects of inhibitors and genetic mutation on the inflammatory pathways versus the fibrogeneic pathways. Two methods were employed to differentiate these effects. The first method showed no significant changes of neutrophils in the bronchoalveolar lavage fluid of Akt-deficient mice and of mice pretreated with PD98059 or SB203580 without or with 5 hours of 30 ml/kg mechanical ventilation. This lack of changes suggests the effects of inhibitors and genetic mutation on the inflammatory process to be less compared with their antifibrotic effects (without ventilation versus with ventilation: Akt group, 1.5 ± 0.2 × 10^5 ^versus 1.7 ± 0.3 × 10^5 ^neutrophils/ml bronchoalveolar lavage; PD98059 group, 1.5 ± 0.3 × 10^5 ^versus 1.6 ± 0.4 × 10^5 ^neutrophils/ml bronchoalveolar lavage; and SB203580 group, 1.6 ± 0.3 × 10^5 ^versus 1.8 ± 0.4 × 10^5 ^neutrophils/ml bronchoalveolar lavage; all *P *< 0.05 versus control). The second method showed an improvement of histopathology indicative of effective re-epithelialization with reduced fibrosis in Akt-deficient mice and in mice with pharmacologic inhibition by PD98059 and SB203580 [18] (Figure 2).

The relative expression of angiogenic and angiostatic factors may alter the extent of pulmonary fibrosis after bleomycin administration. CXC chemokines are characterized by the presence (MIP-2) or by the absence (IP-10) of the Glu-Leu-Arg motif, which dictates their angiogenetic activity in the presence of the Glu-Leu-Arg motif [32]. MIP-2 was involved in induction of acute lung inflammation and was also involved in the pathogenesis of lung fibrosis by regulating angiogenesis, but was independent of fibroblast proliferation [8]. IP-10 has no direct effect on the proliferative activity of pulmonary fibroblasts but regulates deposition of the extracellular matrix by its angiostatic activity, limiting fibroblast migration by epidermal growth factor, by heparin-binding epidermal growth factor-like growth factor, and by platelet-derived growth factor [9]. An imbalance between MIP-2 and IP-10 has been shown to lead to angiogenesis in the pathogenesis of lung fibrosis [8]. We found that high-tidal-volume ventilation increased production of MIP-2 but reduced production of IP-10 (Figure 1c,d). We then went on to examine the pathways involved.

The three MAPKs activated in fibroblasts in the lung tissues of idiopathic pulmonary fibrosis may participate in the fibrogenesis of lung tissue [14]. Akt phosphorylation is elevated in fibroblasts isolated from the lungs of bleomycin-injured mice. In a previous study, inhibition of Akt activity not only alleviated pulmonary inflammation but also alleviated lung damage and fibrinogenic activity [18]. The ERK1/2 pathway may contribute to IL-13-induced remodeling and fibrogenesis, and to MIP-2 production [32]. Reduced hydroxyproline synthesis by inhibition of P38 activation was found in an *in vivo *bleomycin-induced lung fibrosis model in rats [15]. We found that a high tidal volume dose-dependently increased phosphorylation of the Akt and ERK1/2 pathways. Using Akt-deficient mice and an ERK1/2 inhibitor, we found a decrease of lung fibrosis – suggesting the involvement of Akt and ERK1/2 in the regulation of pulmonary fibrosis. Increased phosphorylation of P38 was not found in a dose-independent manner, suggesting the p38 MAPK pathway may have contributed to post-transcriptional induction of MIP-2 and IP-10 syntheses by stabilizing their mRNA via MAPK-activated protein kinase 2 and an adenine-uracil-rich region [33]. High-tidal-volume ventilation did not increase phosphorylation of JNK, suggesting that the JNK pathway was involved in the acute inflammatory lung injury as found in the previous study [13,20].

Members of the S100A4/FSP1 family have been implicated in cytoskeletal–membrane interactions and in cellular growth and differentiation. The expression of S100A4/FSP1 indicates the presence of an ongoing angiogenetic program determining the mesenchymal phenotype [16]. FSP1 is highly specific for fibroblasts but not for the epithelium, mesangial cells, or embryonic endoderm, and is associated with the conversion of epithelial cells to a fibroblast phenotype [17]. In our study, we found that high-tidal-volume ventilation increased positive staining of S100A4/FSP1 in the epithelium and interstitial fibroblasts, indicating the presence of a transition from epithelial cells to fibroblasts (Figure 4).

The physical forces of mechanical ventilation are sensed and converted into the reactions of intracellular signal transduction via stress failure of the plasma membrane, stress failure of the epithelial and endothelial barriers, mechanical stain, or shear stress [34]. Using a rat model of high-tidal-volume ventilation for 1 or 2 hours, other workers have shown that a high tidal volume may lead to an early expression of type III procollagen, the first collagen to be remodeled in the evolution of lung fibrogenesis (used as an early marker of lung parenchyma remodeling) [35,36]. In previous studies using rats (2 hours ventilation at 20 ml/kg V_T_) and hyaluronan synthase knockout mice (5 hours ventilation at 30 ml/kg V_T_), we found high-tidal-volume-induced hyaluronan synthase 3 mRNA and hyaluronan production in fibroblasts, contributing to the extracellular matrix-induced inflammatory changes involved in VILI [37,38]. In this murine model of bleomycin-induced acute lung injury, we found expressions of type I and type III procollagen after 1 hour of high-tidal-volume ventilation (Figure 3), and found microvascular leak and hydroxyproline deposition after 5 hours of high-tidal-volume ventilation.

A variety of cytokines are involved in the process of pulmonary fibrosis, and no one factor is solely responsible for lung fibrosis. While TNFα, IL-1β, transforming growth factor beta, and chemokines (including CCL17, CCL22, CCL2, and CCL3) contribute to the recruitment of inflammatory cells, the altered balance between angiogenic chemokines (CXCL5, CXCL8, and CXCL12) and angiostatic chemokines (CXCL9, CXCL10, and CXCL11) may promote aberrant angiogenesis/fibrosis. All of these mediators induce extracellular matrix deposition by fibroblasts in the early repair process, which aids epithelial migration [18,32]. In our study, we found that high-tidal-volume ventilation increased epithelium–fibroblast transition, collagen accumulation, and MIP-2 production, but deceased production of IP-10.

## Conclusion

**Figure 9:**
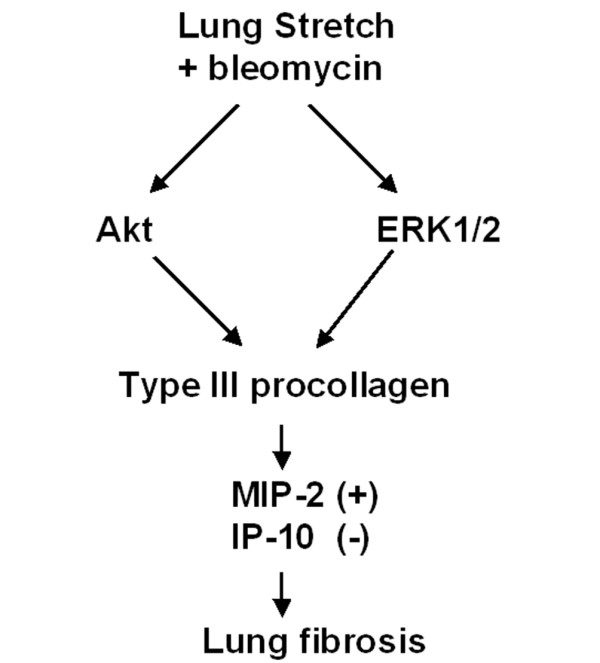
Differences in signaling pathway activation of mechanical ventilation with bleomycin. The serine/threonine kinase/protein kinase B (Akt) and extracellular signal-regulated kinase (ERK) 1/2 pathways were involved in high-tidal-volume ventilation-augmented pulmonary fibrosis after bleomycin-induced acute lung injury, type III procollagen mRNA expression, microvascular permeability, and production of murine macrophage inflammatory protein-2 (MIP-2), but not 10 kDa IFNγ-inducible protein (IP-10) production.

Using an *in vivo *bleomycin mouse model, we have found that high-tidal-volume ventilation increased pulmonary fibrosis by biochemical analysis of hydroxyproline, Masson trichrome staining of collagen, immunohistochemical staining for fibroblasts, microvascular permeability, and production of MIP-2, but not IP-10 production, which was, at least in part, dependent, on the Akt and ERK1/2 pathways (Figure 9). These data have added to the understanding of the effects of mechanical forces in lung fibrosis. In ARDS patients in the early fibroproliferative phase, the inhibition of Akt and ERK1/2 may offer new treatment options.

## Key messages

• High-tidal-volume ventilation increased pulmonary fibrosis in ALI.

• High-tidal-volume ventilation-induced pulmonary fibrosis was dependent on Akt and ERK1/2 activation.

• Inhibition of Akt and of ERK1/2 may offer new treatment options for patients with severe ARDS.

## Abbreviations

Akt = serine/threonine kinase/protein kinase B; ALI = acute lung injury; ARDS = acute respiratory distress syndrome; ERK = extracellular signal-regulated kinase; FSP1 = fibroblast-specific protein 1; GAPDH = glyceraldehyde-phosphate dehydrogenase; IFN = interferon; IL = interleukin; IP-10 = 10 kDa IFNγ-inducible protein; JNK = c-Jun NH_2_-terminal kinase; MAPK = mitogen-activated protein kinase; MIP-2 = murine macrophage inflammatory protein-2; PBS = phosphate-buffered saline; PCR = polymerase chain reaction; RT = reverse transcriptase; TNF = tumor necrosis factor; VILI = ventilator-induced lung injury; V_T _= tidal volume.

## Competing interests

The authors declare that they have no competing interests.

## Authors' contributions

L-KL and DAQ collected and analyzed the data. S-KL, M-JH, and C-CH reviewed and coordinated the study.
